# Codeine and Testosterone Concentration Patterns During Co-Administration of Codeine and Testosterone Undecanoate in Male Wistar Rats

**DOI:** 10.3390/ph19101653

**Published:** 2026-10-20

**Authors:** Murat Ünalmış, Çiğdem Karakükçü, Mükerrem Betül Yerer, Hatice Duman Saraçoğlu

**Affiliations:** 1 Development and Application, Department of Drug Research, Institute of Health Sciences, Erciyes University, Kayseri 38039, Türkiye; 2 Division of Medical Biochemistry, Department of Basic Medical Sciences, Faculty of Medicine, Erciyes University, Kayseri 38039, Türkiye; 3 Department of Pharmacology, Faculty of Pharmacy, Erciyes University, Kayseri 38039, Türkiye

**Keywords:** codeine, testosterone undecanoate, whole-blood codeine, plasma total testosterone, concentration patterns, co-administration, Wistar rat

## Abstract

**Background:** Three widely separated sampling days cannot establish conventional pharmacokinetic parameters. We therefore examined exploratory whole-blood codeine and plasma total-testosterone concentration patterns during co-administration of codeine and testosterone undecanoate in male Wistar rats. **Methods:** Thirty-two rats were allocated to saline (S), codeine (C), testosterone undecanoate (T), or combined codeine plus testosterone undecanoate (CT) groups (eight animals per group). Codeine (10 mg/kg by oral gavage) was administered once daily from Day 0 through Day 5 (six total doses) and testosterone undecanoate (100 mg/kg intramuscularly) on Day 3. On Day 0, whole blood was collected two hours after codeine administration; therefore, Day 0 was a post-dose sampling point rather than a pre-dose baseline. No codeine was administered on Day 6; the Day 6 sample was collected after completion of the six-dose treatment schedule. Codeine dose volumes were adjusted according to each rat’s baseline body weight. Measurements unavailable because of insufficient sample volume were treated as missing without imputation. Codeine was quantified only in C and CT; the corresponding model therefore estimated the association of adding testosterone undecanoate among codeine-treated animals rather than a full factorial drug–drug interaction. **Results:** All 14 available Day 0 samples had instrument-reported whole-blood codeine concentrations (range, 0.339–4.705 ng/mL), consistent with their post-dose status. The C-versus-CT group-by-day test using all instrument-reported values yielded Wald chi-square = 8.487 (df = 2, *p* = 0.014). With the verified LOQ of 0.25 ng/mL, only CT7 Day 14 (0.08 ng/mL) was below the LOQ while remaining above the LOD of 0.07 ng/mL; excluding this observation yielded Wald chi-square = 7.856 (df = 2, *p* = 0.020). Plasma testosterone availability varied by group and day (*n* = 3–8); the raw-scale group-by-day test was *p* = 0.0076, whereas the log-scale sensitivity test was *p* = 0.052. **Conclusions:** The observed numerical patterns are exploratory. Sparse sampling, one below-LOQ codeine value, an influential CT7 observation, unequal plasma-testosterone availability, and sensitivity to analytical and modeling choices prevent causal, pharmacokinetic, metabolic, endocrine-mechanistic, or clinical conclusions.

## 1. Introduction

Codeine is a weak opioid whose activity and safety depend on biotransformation. CYP2D6-mediated O-demethylation forms morphine, CYP3A4-mediated N-demethylation forms norcodeine, and UGT2B7-mediated glucuronidation forms codeine-6-glucuronide. These competing pathways create substantial variation in parent-drug and metabolite profiles [1,2]. A human interaction study that quantified codeine together with its major metabolites further shows that a parent-drug concentration alone cannot identify which metabolic pathway has changed [1].

The intramuscular exposure profile of testosterone undecanoate is formulation-dependent. In rats, a 25 mg/kg formulation study found materially different exposure according to particle size and formulation [3]. This formulation dependence is relevant because the present experiment used a commercial oily product that was manually mixed with saline; exposure equivalence with other testosterone-undecanoate preparations cannot be assumed.

Testosterone and codeine also have a plausible but unverified metabolic intersection. An in vitro study reported competitive inhibition of human UGT2B7 by testosterone, with Ki values of 11.4 micromolar in a recombinant system and 30.2 micromolar in pooled human liver microsomes [4]. It did not test testosterone undecanoate, codeine, rats, or an in vivo interaction, and the authors considered systemic hepatic modulation unlikely at ordinary circulating testosterone concentrations. The study therefore supports a testable hypothesis only, not causal attribution in the present experiment.

Previous studies in male Wistar rats used intramuscular testosterone undecanoate at 100 mg/kg, including daily administration for one week [5] and a separate single-dose Nebido regimen [6]. Another experimental study reported intramuscular administration of Nebido after dilution with 0.9% NaCl in mice [7]. These reports provide experimental precedent for the dose and route and for saline dilution, respectively, but they do not establish exposure comparability or validate the exact preparation ratio used in the present study. The 100 mg/kg dose used here represents a very high experimental exposure and should not be interpreted as equivalent to a clinical replacement regimen.

Opioid exposure can suppress the hypothalamic–pituitary–gonadal axis, and a systematic review and meta-analysis found frequent gonadal-axis suppression among predominantly male populations receiving chronic opioids [8]. Those clinical data do not establish an endocrine effect of short-term codeine exposure in rats, but they provide a rationale for measuring plasma total testosterone together with whole-blood codeine. The exploratory question in this study was whether the available whole-blood codeine and plasma total-testosterone concentration patterns differed across treatment groups and the three sampling days. Because LH, FSH, estradiol, metabolites, UGT activity, and conventional pharmacokinetic parameters were not measured, the study cannot establish endocrine-axis suppression, metabolic competition, or a pharmacokinetic drug–drug interaction.

## 2. Results

### 2.1. Animal Disposition, Analytical Availability, and Interpretability

All 32 animals completed the protocol; no deaths, adverse events, or study exclusions occurred. Some analyses could not be performed because sample volume was insufficient. Whole-blood codeine measurements were available only for animals 2–8 in C and CT (*n* = 7 per group on each day), with no measurements for C1 or CT1. All 14 available Day 0 samples had instrument-reported numerical codeine concentrations (range, 0.339–4.705 ng/mL). Day 0 whole-blood collection occurred two hours after codeine administration; therefore, these values are post-dose measurements and must not be described as pre-dose or baseline signals. Sample identifiers were checked, and review of the available laboratory records did not identify evidence of instrument carry-over, contamination, or sample switching. Plasma testosterone availability on Days 0/6/14 was S 7/3/8, C 4/5/8, T 6/3/8, and CT 5/6/8. Missing measurements were not converted to zero or imputed, but the available records were insufficient to establish an ignorable missingness mechanism.

### 2.2. Body Weight

Initial recorded body weights ranged from 225 to 328 g. Baseline weights did not differ significantly among groups (F (3, 28) = 1.182, *p* = 0.334), and Day 14 weights also did not differ (F (3, 28) = 0.685, *p* = 0.569), although Day 14 variance heterogeneity was detected (median-centered Levene *p* = 0.034). Weight change differed among groups (F (3, 28) = 5.158, *p* = 0.0058, eta-squared = 0.356). In exploratory pooled-error pairwise comparisons with Holm adjustment, only C versus T remained significant (difference = 11.875 g, Holm *p* = 0.012, Hedges g = 1.70). Body-weight changes by treatment group are summarized in Table 1.

### 2.3. Longitudinal Whole-Blood Codeine Concentrations

The codeine analysis addressed a restricted estimand: the difference in three-day numerical concentration patterns between C and CT, corresponding to the association of adding testosterone undecanoate among codeine-treated animals. Whole-blood codeine was not quantified in S or T, so this was not a full 2 × 2 factorial drug-interaction test. Using all instrument-reported numerical values, the nominal group-by-day Wald test was chi-square = 8.487 (df = 2, *p* = 0.014). No prospective rule for handling below-LOQ observations was documented. With the corrected LOQ of 0.25 ng/mL, only one measurement (CT7 Day 14, 0.08 ng/mL) was below the LOQ while remaining above the verified LOD of 0.07 ng/mL. Excluding this observation in a post hoc sensitivity analysis yielded chi-square = 7.856 (df = 2, *p* = 0.020). These analyses do not establish a difference in AUC, clearance, Cmax, Tmax, half-life, or a pharmacokinetic drug–drug interaction. The available-case whole-blood codeine summaries are provided in Table 2.

The Holm-adjusted CT-minus-C contrast was significant only on Day 6 (difference = 3.027 ng/mL, 95% CI 0.732 to 5.323; raw *p* = 0.0097; Holm *p* = 0.029 across three days). The Day-6-versus-Day-0 difference-in-change contrast was 3.585 ng/mL (95% CI 0.339 to 6.831; raw *p* = 0.030; Holm *p* = 0.061 across two interaction contrasts).

**Figure 1:**
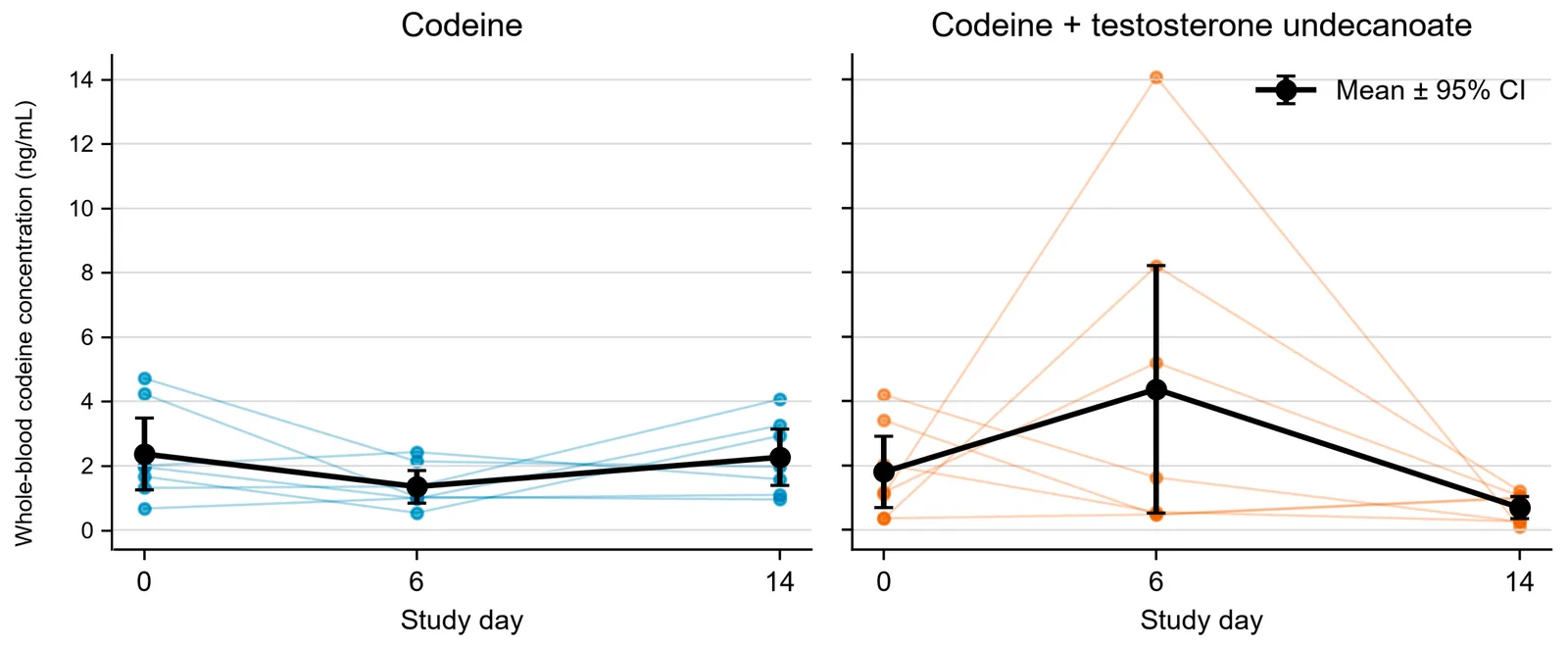
Individual instrument-reported whole-blood codeine concentrations and group means with approximate 95% confidence intervals on Days 0, 6, and 14. Seven animals were available per group and day. CT7 Day 14 (0.08 ng/mL) was below the verified LOQ of 0.25 ng/mL but above the LOD of 0.07 ng/mL and is displayed as reported. Lines are visual guides and do not represent dense pharmacokinetic profiles. Light blue and orange lines with small points represent individual animals in C and CT, respectively; black points and connecting lines represent group means, and error bars represent approximate 95% confidence intervals. Both panels share the same y-axis scale; therefore, the right panel does not repeat the y-axis title and tick labels. C, codeine; CT, combined codeine plus testosterone undecanoate.

CT7 had a Day 6 whole-blood codeine concentration of 14.067 ng/mL. The CT7 Day 6 specimen was reanalyzed in duplicate in the same run sequence, and the elevated concentration was confirmed. With CT7 included, the group-by-day interaction was Wald chi-square = 8.487 (df = 2, *p* = 0.014). Removing only the CT7 Day 6 observation yielded Wald chi-square = 6.810 (df = 2, *p* = 0.033), whereas removing all three CT7 observations yielded Wald chi-square = 5.877 (df = 2, *p* = 0.053). CT7 is therefore influential despite the duplicate confirmation, and the finding remains exploratory. Individual animal profiles and group summaries are shown in Figure 1; the underlying individual values and profiles are also provided in Figures S1 and S2 and Tables S1 and S2.

### 2.4. Plasma Total-Testosterone Concentrations

The available-case raw-scale group-by-day test yielded Wald chi-square = 17.514 (df = 6, *p* = 0.0076), whereas the log-scale sensitivity test yielded chi-square = 12.476 (df = 6, *p* = 0.052). Eighteen plasma testosterone observations were below 100 pg/mL. Replacing them with 100 pg/mL yielded chi-square = 18.511 (df = 6, *p* = 0.0051), and replacing them with 0.07 ng/mL yielded chi-square = 17.941 (df = 6, *p* = 0.0064). Thus, the nominal raw-scale group-by-day result was retained under both substitution rules, but the log-scale result crossed the 0.05 threshold. Random-intercept estimates were at or near the boundary. Availability was markedly unequal, particularly on Day 6, and the missingness mechanism could not be established from the available records. These analyses describe plasma total-testosterone concentration patterns only and do not demonstrate hypothalamic–pituitary–gonadal-axis suppression or another endocrine mechanism. Available-case plasma total-testosterone summaries are provided in Table 3.

**Figure 2:**
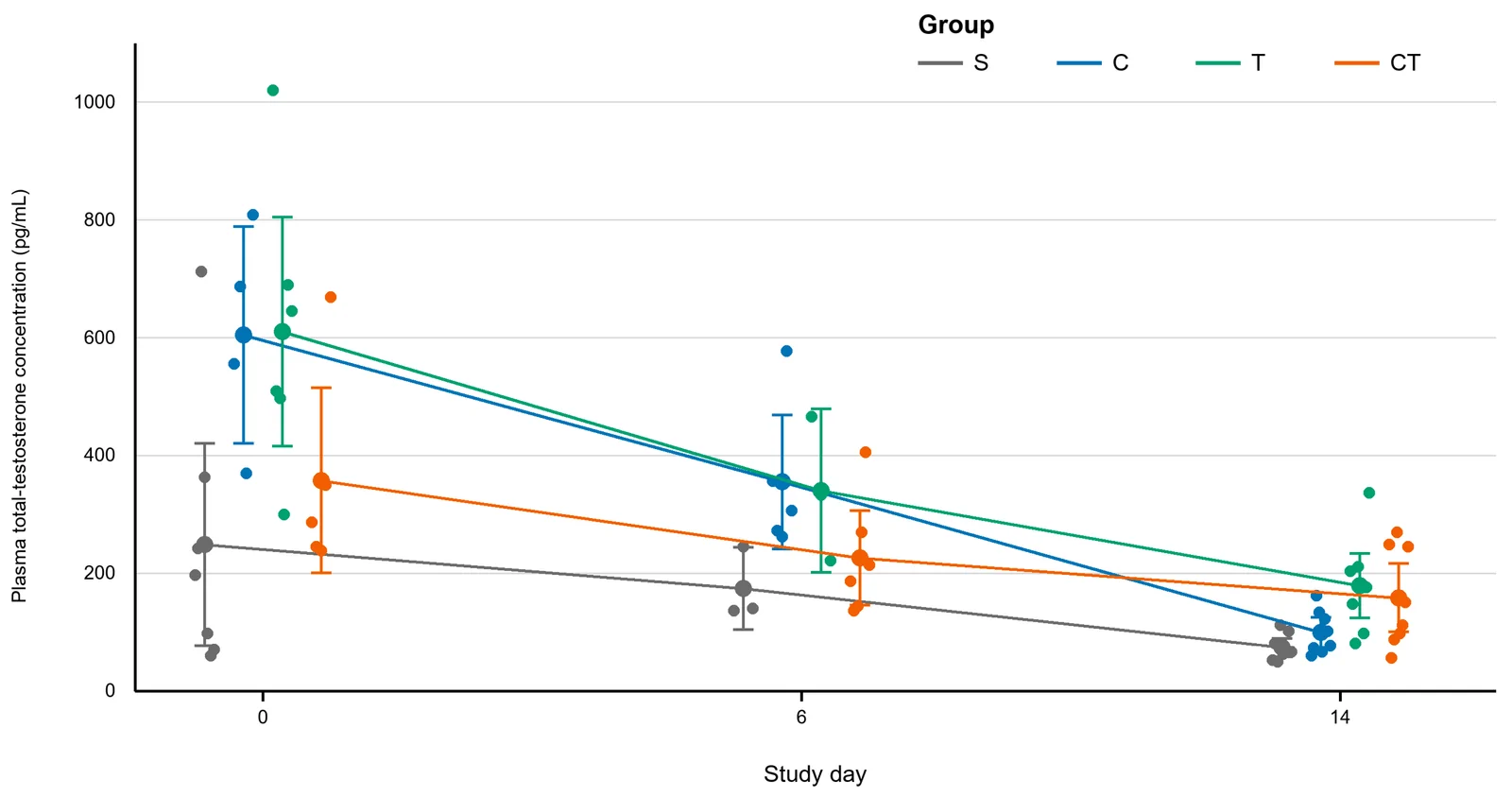
Individual plasma total-testosterone concentrations and available-case means with approximate 95% confidence intervals. Available *n* on Days 0/6/14 was S 7/3/8, C 4/5/8, T 6/3/8, and CT 5/6/8. Colored small points represent individual observations, larger points and connecting lines represent available-case group means, and error bars represent approximate 95% confidence intervals; colors distinguish treatment groups as indicated in the legend. S, saline; C, codeine; T, testosterone undecanoate; CT, combined codeine plus testosterone undecanoate.

In raw-scale Holm-adjusted difference-in-change contrasts versus S, C differed at Day 14 versus Day 0 (−330.97 pg/mL, 95% CI −527.97 to −133.97; Holm *p* = 0.006), as did T (−257.17 pg/mL, 95% CI −440.72 to −73.61; Holm *p* = 0.030). Corresponding Day 6 and CT-versus-S contrasts were not significant after adjustment. These contrasts do not override the log-scale sensitivity result. Individual observations and available-case group means are shown in Figure 2.

## 3. Discussion

The whole-blood codeine data provide instrument-reported numerical patterns at three widely separated sampling days, not a conventional pharmacokinetic profile. Day 0 was a post-dose sampling point: whole blood was collected two hours after codeine administration. Sample identifiers were checked, and the available laboratory review did not identify evidence of instrument carry-over, contamination, or sample switching. The verified analytical method used a rat whole-blood calibration range of 3.25–30 ng/mL, an LOD of 0.07 ng/mL, and an LOQ of 0.25 ng/mL. Concentrations between the LOQ and the lowest calibrator were estimated by downward extrapolation only when chromatographic peak integrity and the required signal-to-noise criterion were satisfied. Exposure, clearance, and metabolites were not measured, and parent-codeine values alone cannot identify whether CYP- or UGT-dependent pathways changed [1]. The study therefore does not demonstrate a pharmacokinetic interaction or a UGT-mediated mechanism.

The nominal codeine result was sensitive to both the single below-LOQ observation and CT7. Excluding CT7 Day 14 (0.08 ng/mL) changed the interaction from *p* = 0.014 to *p* = 0.020. The elevated CT7 Day 6 value was confirmed by duplicate reanalysis in the same run sequence. With CT7 included, the interaction was *p* = 0.014; removing that Day 6 observation yielded *p* = 0.033, and removing all three CT7 observations yielded *p* = 0.053. Every available Day 0 sample had a numerical whole-blood codeine concentration because blood was collected two hours after codeine administration. This post-dose timing explains why parent codeine was measurable on Day 0 but does not convert the three sampling days into a conventional pharmacokinetic profile.

Plasma total-testosterone concentrations declined numerically across all groups; however, statistical inference was scale-sensitive, Day 6 sample sizes were small, and data missingness was unequal. Although the nominal raw-scale group-by-day interaction remained significant after substituting values below 100 pg/mL with either 100 or 70 pg/mL, the log-scale sensitivity analysis yielded *p* = 0.052. Furthermore, mechanistic markers—including LH, FSH, estradiol, corticosterone, and ACTH—were not evaluated, precluding the differentiation between opioid-mediated hypothalamic–pituitary suppression, androgen feedback, peripheral aromatization, and procedural stress.

In addition to these physiological and statistical constraints, assay-related limitations must be considered. The MAGLUMI Testosterone assay was utilized based on the highly conserved chemical structure of testosterone across mammals and its prior application in male rat models [9]. Nonetheless, the assay is commercially intended for human serum, and formal species- or matrix-specific analytical validation in rat plasma was not conducted herein. Given the potential for uncharacterized matrix effects alongside the missing mechanistic parameters, these testosterone findings should be interpreted as exploratory relative trends rather than definitive evidence of endocrine disruption or fully validated absolute concentrations [10,11].

The exact testosterone-undecanoate formulation matters [3]. Prior male-Wistar studies used 100 mg/kg intramuscular testosterone undecanoate as daily dosing for one week [5] and as a single-dose Nebido regimen [6]. A published mouse study also reported intramuscular Nebido after dilution with 0.9% NaCl [7]. These studies provide experimental precedent, but they do not establish exposure comparability or validate the exact preparation ratio used in the present single-dose experiment. The 100 mg/kg dose used here was a very high experimental exposure and is not comparable with clinical Nebido administration or testosterone-replacement dosing. The commercial oily product was manually mixed with saline without compatibility, phase-homogeneity, stability, or delivered-dose testing. The actual uniformity of exposure between animals therefore cannot be established.

The in vitro UGT study supports only mechanistic plausibility [4]. It used testosterone rather than testosterone undecanoate, human systems rather than rats, and probe substrates rather than codeine. The present experiment cannot connect its whole-blood codeine and plasma testosterone patterns to the reported Ki values.

### Limitations

This study has several experimental, statistical, and analytical limitations. Experimentally, the study was limited by small, male-only cohorts sampled across three timepoints, a lack of prospectively documented power calculations, an unvalidated oil–saline vehicle for the supraphysiological testosterone undecanoate dose, non-matched oral and intramuscular handling procedures, unmodeled cage clustering, and the omission of key mechanistic biomarkers (LH, FSH, estradiol, metabolites, and enzyme kinetics). Statistically, insufficient sample volume caused unequal, potentially non-ignorable missingness, resulting in scale-sensitive inference, boundary random-effect estimates, and an influential CT7 observation despite duplicate reanalysis. Analytically, although testosterone’s structure is conserved across mammals and the immunoassay has prior precedent in male rats, the lack of species- and matrix-specific validation in rat plasma—together with observations falling below the lower reporting limit—precludes full confidence in analytical accuracy.

## 4. Materials and Methods

### 4.1. Ethics, Animals, Housing, Allocation, and Masking

The protocol was approved by the Erciyes University Animal Experiments Local Ethics Committee (HADYEK; approval 23/080; 5 April 2023). Thirty-two male Wistar rats aged 8–12 weeks were obtained from DEKAM and acclimatized for seven days. Recorded initial body weights ranged from 225 to 328 g. Animals were housed four per standard cage on wood-shaving bedding at approximately 21 degrees C under a 12 h light/12 h dark cycle with a standard pellet diet and water ad libitum. Relative humidity was not recorded numerically; enrichment was not provided. Food and water consumption were not measured.

Animals were individually marked using tail pen/paint. The first author performed manual allocation with DEKAM personnel present: animals were numbered, identifiers were selected without viewing them, and animals were assigned sequentially to S, C, T, or CT (eight per group). Each group occupied two group-labeled cages with four animals per cage throughout the experiment. Cage was not modeled as a clustering factor.

The first author knew group identity during dosing. Samples were sent to the laboratory under codes such as S1, C2, T2, or CT3; laboratory personnel saw these codes but were not given the treatment decoding key. Group identity was known during statistical analysis. The study was therefore not fully blinded.

Animals were observed daily by the first author and DEKAM personnel during routine husbandry. No study-specific welfare scoring sheet or prespecified intervention thresholds were used. No deaths, adverse events, or exclusions occurred. Eight animals per group were specified in the approved protocol; a numerical prospective power-analysis record was not available.

### 4.2. Treatments and Timeline

The S group received 0.2 mL sterile 0.9% sodium chloride solution (Polifleks; Polifarma İlaç San. ve Tic. A.Ş., Ergene, Tekirdağ, Türkiye) by oral gavage once daily from Day 0 through Day 5. The C and CT groups received codeine sulfate salt (batch no. TR AAF 170818 ARG/17-01 KS; Turkish Grain Board, Opium Alkaloids Factory Plant Management, Bolvadin, Afyonkarahisar, Türkiye) at 10 mg/kg by oral gavage using a rounded metal rat gavage needle once daily from Day 0 through Day 5, for a total of six doses. On Day 0, whole blood was collected two hours after codeine administration; thus, the Day 0 sample was post-dose rather than a pre-dose baseline. No codeine was administered on Day 6; the Day 6 sample was obtained after completion of the six-dose treatment schedule, following the sixth and final dose on Day 5. Each codeine dose volume was calculated according to the individual rat’s baseline body weight. The confirmed dosing schedule comprised one dose daily on Days 0, 1, 2, 3, 4, and 5 (six total doses). One 10 mg/mL batch was prepared by dissolving 360 mg codeine sulfate in 36 mL 0.9% saline and was refrigerated during the dosing period. The exact refrigerator temperature was not recorded, and no stability test was performed.

Nebido (testosterone undecanoate 250 mg/mL; Bayer, Leverkusen, Germany; lot KT0LVKL) was obtained from a pharmacy. A nominal 100 mg/mL preparation was made by combining 2.4 mL Nebido with 3.6 mL 0.9% saline, mixing by manual shaking, and using it immediately. No formal compatibility, homogeneity, or stability assessment was performed. T and CT received one 100 mg/kg intramuscular dose in one hind leg on Day 3, calculated from baseline body weight (approximately 1 mL/kg), using a 1 mL insulin syringe (Berika Teknoloji Medikal, Konya, Türkiye). Exact side, muscle, and needle gauge/length were not recorded. On Day 3, gavage preceded testosterone administration by at least one hour; the exact interval was not recorded.

**Figure 3:**
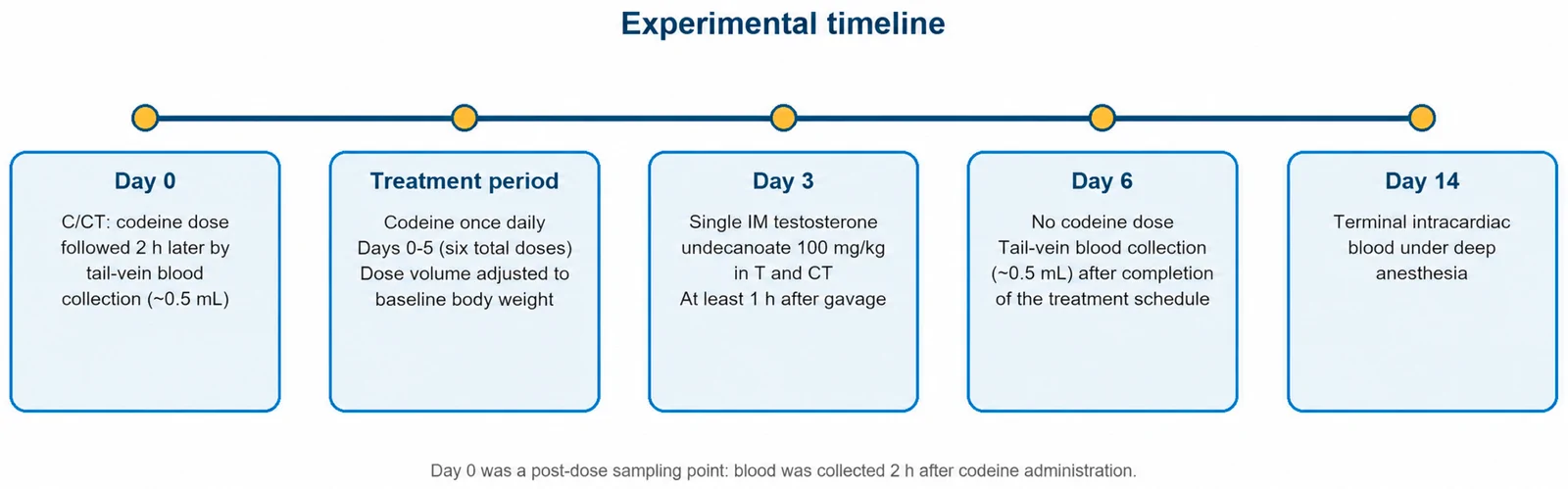
Experimental treatment and blood-sampling timeline. The wavy line denotes the passage of time between the labeled study events. IM, intramuscular; C, codeine; T, testosterone undecanoate; CT, combined treatment.

T received testosterone undecanoate without oral saline, whereas S and C did not receive a sham intramuscular injection. The control procedures were therefore not vehicle- or handling-matched. Consequently, comparisons involving testosterone undecanoate cannot fully separate the nominal drug exposure from injection, vehicle, and handling effects. Day 3 placed androgen exposure within the codeine treatment schedule; no alternative timing was tested, and the timing was not justified as a rat half-life-based optimum. The treatment and blood-sampling schedule is summarized in Figure 3.

### 4.3. Blood Collection and Sample Handling

Blood was sampled between approximately 06:00 and 10:00. Days 0 and 6 used approximately 0.5 mL nonterminal tail-vein blood; no codeine was administered on Day 6, and the Day 6 sample was obtained after completion of the six-dose schedule following the final Day 5 dose. Day 14 used terminal intracardiac collection. Sampling was performed under Ketamin 10% (100 mg/mL; Dutch Farm International B.V., Nederhorst den Berg, The Netherlands) at 60 mg/kg plus Xylazin Bio 2% (20 mg/mL; Bioveta a.s., Ivanovice na Hané, Czech Republic) at 10 mg/kg, administered intraperitoneally. Fasting status could not be established from contemporaneous records and is not asserted.

For terminal sampling, intracardiac blood collection/exsanguination was performed under deep anesthesia, and death was confirmed by cessation of respiration and heartbeat. Cervical dislocation was not performed.

Blood samples were collected into EDTA-containing tubes (whole blood for codeine analysis and plasma for testosterone analysis). Plasma samples were obtained by centrifugation at 2500× *g* for 10 min. Both whole blood and plasma samples were aliquoted into Eppendorf tubes and stored at −20 °C until the day of analysis. Samples were thawed once and used immediately without refreezing.

### 4.4. Codeine Measurement by LC-MS/MS

#### 4.4.1. Instrumentation and Analytical Method

Codeine concentrations in whole blood samples were measured using an Agilent 1260 Infinity II liquid chromatography system coupled to an Agilent 6475 triple quadrupole mass spectrometer (LC-MS/MS; Agilent Technologies, Santa Clara, CA, USA). Chromatographic separation was performed using a JASEM JSM-CL-4075 C18 column (150 × 4.6 mm, 2.7 μm; SEM Laboratuar Cihazları, Istanbul, Türkiye) maintained at 40 °C. Mobile phase A consisted of 2 mM ammonium acetate (Supelco, Bellefonte, PA, USA) in water, while mobile phase B was LC–MS-grade methanol (Supelco, Merck KGaA, Darmstadt, Germany). The flow rate was set at 0.70 mL/min, the injection volume was 10 μL, and the total run time was 14 min. The gradient program started with 10% mobile phase B (90% A) and was maintained for 0.30 min. Mobile phase B was then increased linearly from 10% to 80% between 0.30 and 3.00 min, followed by a linear increase to 95% between 3.00 and 7.00 min. The mobile phase was maintained at 95% B until 9.00 min and subsequently returned to 10% B at 9.10 min for column re-equilibration until the end of the run.

Mass spectrometric detection was performed using a heated electrospray ionization (ESI) source operated in positive ionization mode. Codeine-d3 (100 ng/mL) was used as the deuterated internal standard. Two multiple reaction monitoring (MRM) transitions were monitored for codeine and codeine-d3. The corresponding MRM transitions, optimized collision energies, and retention times are presented in Table 4. A dwell time of 2 ms was used for each MRM transition. Data acquisition and quantitative analysis were performed using Agilent MassHunter Quantitative Analysis, version 12.1 (build 12.1.938.3; Agilent Technologies, Santa Clara, CA, USA), and spectral confirmation was performed using the MassHunter Mass Spectral Library.

For sample preparation, 50 μL of whole blood was mixed with 15 μL of the internal standard solution and 200 μL of a 50:50 (*v*/*v*) mixture of LC–MS-grade acetonitrile (Sigma-Aldrich, St. Louis, MO, USA) and methanol. The mixture was vortexed for 30 s, sonicated in an ultrasonic bath for 10 min, and vortexed again for 30 s. Samples were then centrifuged at 14,000 rpm for 5 min. The resulting supernatants were transferred into autosampler vials and analyzed by LC-MS/MS.

**Figure 4:**
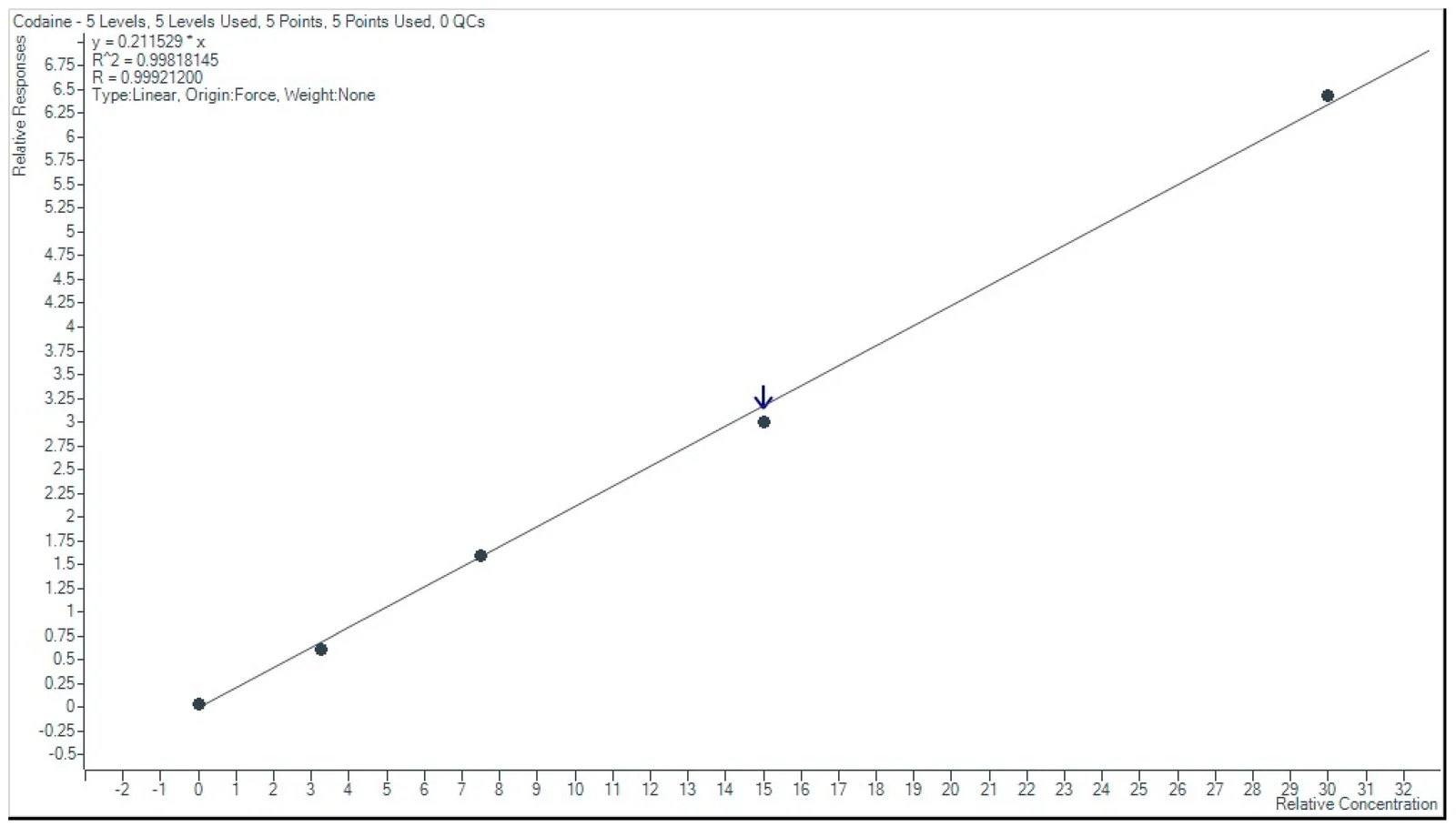
Calibration curve generated for the analytical run. Filled circles represent the five non-zero calibration points used in the regression; the solid line represents the unweighted linear fit forced through the origin (y = 0.211529x; R^2^ = 0.99818145; r = 0.99921200). The blue arrow is the software selection marker for the highlighted 15 ng/mL calibration point and has no analytical significance.

Calibration was performed using five non-zero nominal working-standard concentrations (1.625, 3.25, 7.5, 15, and 30 ng/mL) together with a zero calibrator. The zero calibrator was excluded from the weighted calibration regression. Calibration curves were generated by unweighted linear regression forced through the origin. The final accepted curve was y = 0.211529x (R^2^ = 0.99818145; r = 0.99921200; Figure 4). Two quality control (QC) levels, corresponding to 4 and 16 ng/mL, were included in each analytical run. The acceptance criterion for QC samples was set at ±20% of the nominal concentration. In the analytical run, the measured concentrations were 4.17 ng/mL and 16.67 ng/mL for the 4 and 16 ng/mL QC levels, corresponding to biases of +4.25% and +4.19%, respectively. Both QC levels met the predefined acceptance criteria, and study samples were subsequently analyzed. Samples with codeine concentrations between the method LOQ (0.25 ng/mL) and the lowest calibrator (1.625 ng/mL) were calculated by downward extrapolation of the weighted calibration curve, provided that the chromatographic signal-to-noise ratio satisfied the LOQ criterion (S/N ≈ 10).

**Figure 5:**
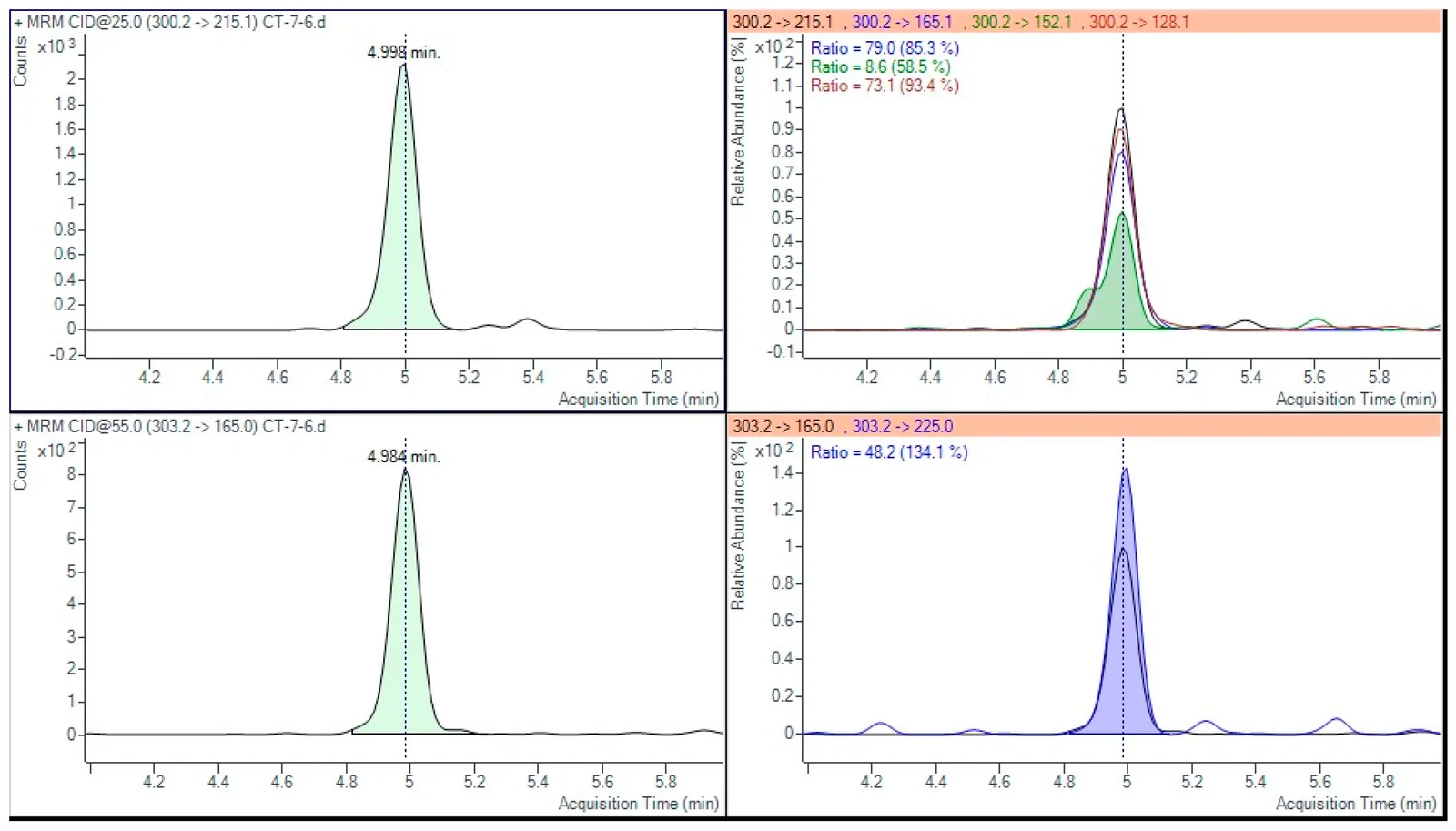
Representative LC–MS/MS chromatograms for codeine (**upper row**) and codeine-d3 (**lower row**) in the CT7 Day 6 specimen. The left panels show the quantifier chromatograms, and the right panels show the overlaid monitored ion transitions and their relative abundances. Colored traces correspond to the transitions identified in the panel legends, and the shaded areas indicate the integrated peaks.

The representative chromatograms of codeine and codeine-d3 are shown in Figure 5.

#### 4.4.2. Method Verification for Rat Whole Blood

The applicability of the method to rat whole blood was verified by evaluating calibration performance, accuracy, precision, matrix effect, carry-over, limit of detection (LOD), and limit of quantification (LOQ). Calibration was assessed over the range of 1.625–30 ng/mL using five concentration levels. Accuracy and precision were evaluated at two calibration levels (LQC, 3.25 and HQC, 15 ng/mL), with three replicates at each level. Accuracy was expressed as percentage bias, and precision as the coefficient of variation (CV%). Acceptance criteria were set at ±20% for accuracy and ≤20% for precision.

The matrix effect was assessed at the same two concentration levels using rat whole blood. The matrix factor (MF) was calculated by comparing the peak areas of codeine in post-extraction spiked matrix samples with those of corresponding neat standard solutions at the same concentrations. The internal-standard-normalized matrix factor was calculated by normalizing the matrix factor of codeine to that of codeine-d3.

The LOD and LOQ were determined based on the signal-to-noise (S/N) approach, using S/N ratios of approximately 3 and 10, respectively. Carry-over was assessed by injecting a blank rat whole-blood sample following the highest calibration standard (30 ng/mL). The complete method-verification results are summarized in Table 5.

### 4.5. Testosterone Measurement

Plasma total testosterone was measured on a Maglumi 2000 analyzer using the competitive MAGLUMI Testosterone chemiluminescence immunoassay (catalog no. 130202010M; Snibe Diagnostic, Shenzhen, China), which uses anti-testosterone magnetic microbeads and an ABEI conjugate. The assay information supplied by the study laboratory indicated a reportable range of 100–20,000 pg/mL, and the manufacturer specifies human serum as the intended specimen [12].

The assay was selected based on the highly conserved chemical structure of steroid hormones, including testosterone, across mammalian species, as well as previous use of the same MAGLUMI Testosterone assay for testosterone measurement in male rats [9].

The analytical dataset included numerical testosterone results below the stated lower reporting boundary of 100 pg/mL. These values were retained as reported by the analyzer for the primary exploratory analysis rather than being replaced by the reporting threshold. To evaluate the potential influence of this approach, sensitivity analyses were additionally performed by replacing values below 100 pg/mL with either 100 pg/mL or an alternative substitution value of 70 pg/mL. The results of these sensitivity analyses were compared with those of the original analysis to assess whether the handling of low-level observations materially affected the statistical findings.

### 4.6. Statistical Analysis

Reproducible analyses were performed in Python 3.12.13 using NumPy 2.5.2, pandas 3.0.5, SciPy 1.18.0, and statsmodels 0.14.6. Continuous outcomes are summarized as mean +/- SD with exact available n. Maximum-likelihood linear mixed-effects models included categorical group, categorical day, and group-by-day interaction as fixed effects and animal as a random intercept. The codeine model was restricted to C and CT because whole-blood codeine was quantified only in the two codeine-treated groups; its estimand was the association of adding testosterone undecanoate among codeine-treated animals, not a full factorial drug-by-drug interaction. Plasma total testosterone was evaluated across all four groups. Fixed-effect joint tests used Wald chi-square statistics, and random-intercept estimates were checked for boundary behavior.

Model-based contrasts were reported with 95% confidence intervals, and *p* values were Holm-adjusted within the stated contrast family. No prospective analysis record specified the primary treatment of below-LOQ codeine values. The analysis using supplied numerical values and exclusion of the single value below the corrected LOQ are therefore presented as post hoc sensitivity analyses without designating either as confirmatory. Additional post hoc codeine sensitivities omitted CT7’s Day 6 value or the complete CT7 record. Testosterone sensitivities used log-transformed concentrations and substitution of values below 100 pg/mL with 100 or 70 pg/mL. Body weights were examined by one-way ANOVA; exploratory pooled-error pairwise comparisons were Holm-adjusted and accompanied by Hedges g. Median-centered Levene tests assessed variance homogeneity. Two-sided *p* < 0.05 was used, but all inferential results are interpreted as exploratory because no contemporaneous confirmatory multiplicity plan or numerical power-analysis record was available.

## 5. Conclusions

The supplied data show exploratory whole-blood codeine and plasma total-testosterone concentration patterns in male Wistar rats. They do not establish a treatment effect or drug–drug interaction because sampling was sparse, one codeine value was below the verified LOQ, CT7 was influential despite duplicate confirmation, plasma-testosterone availability was unequal, and formulation and control-matching limitations remained. Day 0 sampling occurred two hours after codeine administration and therefore represents a post-dose observation rather than a pre-dose baseline; no codeine was administered on Day 6, and the Day 6 sample was obtained after completion of the six-dose schedule following the final Day 5 dose. The study does not support conclusions about total exposure, conventional pharmacokinetics, UGT competition, endocrine-axis suppression, or clinical consequences. Confirmation requires complete batch-level analytical documentation, validated formulations, matched vehicle and handling controls, prospectively justified sample sizes, dense parent-drug and metabolite sampling, direct mechanistic measurements, cage-aware designs, and experiments in both sexes.

## Figures and Tables

**Table 1:** Body-weight change by treatment group.

Group	*n*	Mean Change (g)	SD (g)
S	8	8.625	5.579
C	8	18.000	7.616
T	8	6.125	5.410
CT	8	15.500	8.734

Abbreviations: S, saline; C, codeine; T, testosterone undecanoate; CT, combined codeine plus testosterone undecanoate; *n*, number of animals; SD, standard deviation.

**Table 2:** Available-case whole-blood codeine concentrations (ng/mL), summarized from instrument-reported numerical values, including the single value below the verified LOQ.

Group	Day	*n*	Mean	SD
C	0	7	2.353	1.517
C	6	7	1.339	0.683
C	14	7	2.250	1.177
CT	0	7	1.796	1.499
CT	6	7	4.366	5.195
CT	14	7	0.688	0.468

Abbreviations: C, codeine; CT, combined codeine plus testosterone undecanoate; *n*, number of animals; SD, standard deviation; LOQ, limit of quantification.

**Table 3:** Available-case plasma total-testosterone concentrations (pg/mL).

Group	Day 0, *n*; Mean +/- SD	Day 6, *n*; Mean +/- SD	Day 14, *n*; Mean +/- SD
S	7; 248.797 +/- 231.472	3; 174.158 +/- 61.622	8; 73.989 +/- 22.471
C	4; 605.187 +/- 187.923	5; 355.046 +/- 129.458	8; 99.408 +/- 36.672
T	6; 610.474 +/- 242.963	3; 340.143 +/- 122.628	8; 178.498 +/- 79.075
CT	5; 357.790 +/- 179.606	6; 225.956 +/- 100.477	8; 158.469 +/- 83.972

Abbreviations: S, saline; C, codeine; T, testosterone undecanoate; CT, combined codeine plus testosterone undecanoate; *n*, number of animals; SD, standard deviation.

**Table 4:** MRM transitions and compound-dependent parameters for codeine and codeine-d3.

Analyte	Precursor *m*/*z*	Product *m*/*z*	CE (V)	RT	Transition Role
Codeine	300.20	215.10	25	4.998	Quantifier
	300.20	165.10	25	4.998	Qualifier
Codeine-d3	303.20	165.00	55	4.984	Quantifier
	303.20	225.00	29	4.984	Qualifier

Abbreviations: *m*/*z*, mass-to-charge ratio; CE, collision energy; RT, retention time.

**Table 5:** Verification results for the determination of codeine in rat whole blood by LC-MS/MS.

Verification Parameter	Acceptance Criterion	Result
**Calibration**	R^2^ > 0.99	0.9981
**Accuracy**	Bias within ±20%	LQC: −14% HQC: 2.4%
**Precision**	CV ≤ 20%	LQC: 5.9% HQC: 1.3%
**Matrix** **E** **ffect**	80–120%	113%
**LOD**	S/N ≈ 3	0.07 ng/mL
**LOQ**	S/N ≈ 10	0.25 ng/mL
**Carry-over**	No significant peak at codeine retention time	No

Abbreviations: R^2^, coefficient of determination; LQC, low-concentration quality control; HQC, high-concentration quality control; CV, coefficient of variation; LOD, limit of detection; LOQ, limit of quantification; S/N, signal-to-noise ratio.

## Data Availability

The original contributions presented in this study are included in the article/Supplementary Material. Further inquiries can be directed to the corresponding authors.

## Supplementary Materials

The following supporting information can be downloaded at https://www.mdpi.com/article/10.3390/ph19101653/s1, Figure S1. Individual instrument-reported whole-blood codeine profiles in the codeine group (C), *n* = 7 animals with available values on Days 0, 6, and 14. Concentrations are ng/mL. No C value was below the verified 0.25 ng/mL LOQ; Figure S2. Individual instrument-reported whole-blood codeine profiles in the combined codeine plus testosterone undecanoate group (CT), *n* = 7 animals with available values on Days 0, 6, and 14. Concentrations are ng/mL. One CT measurement (CT7 Day 14, 0.081188 ng/mL) was below the verified 0.25 ng/mL LOQ but remained above the 0.07 ng/mL LOD and is displayed as reported; Table S1. Available individual whole-blood codeine concentrations; Table S2. Available individual plasma total-testosterone concentrations.
