# Supplementary material for: Codeine and Testosterone Concentration Patterns During Co-Administration of Codeine and Testosterone Undecanoate in Male Wistar Rats
Source: Pharmaceuticals (Basel). 2026 Oct 20;19(10):1653. doi: 10.3390/ph19101653 (PMC13708289; doi:10.3390/ph19101653)
Supplement: Supplementary file 1 [file pharmaceuticals-19-01653-s001.zip › pharmaceuticals-4590955-supplementary.pdf]

Supplementary Materials

Codeine and Testosterone Concentration Patterns during Co-administration of Codeine and Testosterone Undecanoate in Male Wistar Rats

Supplementary Figures

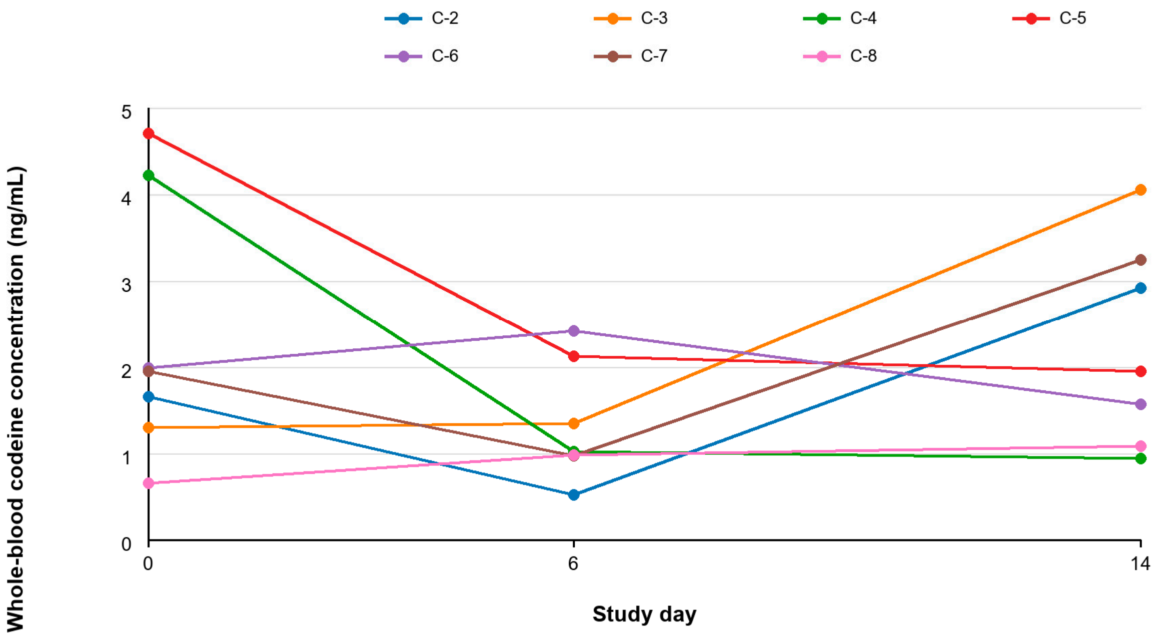

Figure S1. Individual instrument-reported whole-blood codeine profiles in the codeine group (C), n = 7 animals with available values on Days 0, 6, and 14. Concentrations are ng/mL. No C value was below the verified 0.25 ng/mL LOQ.

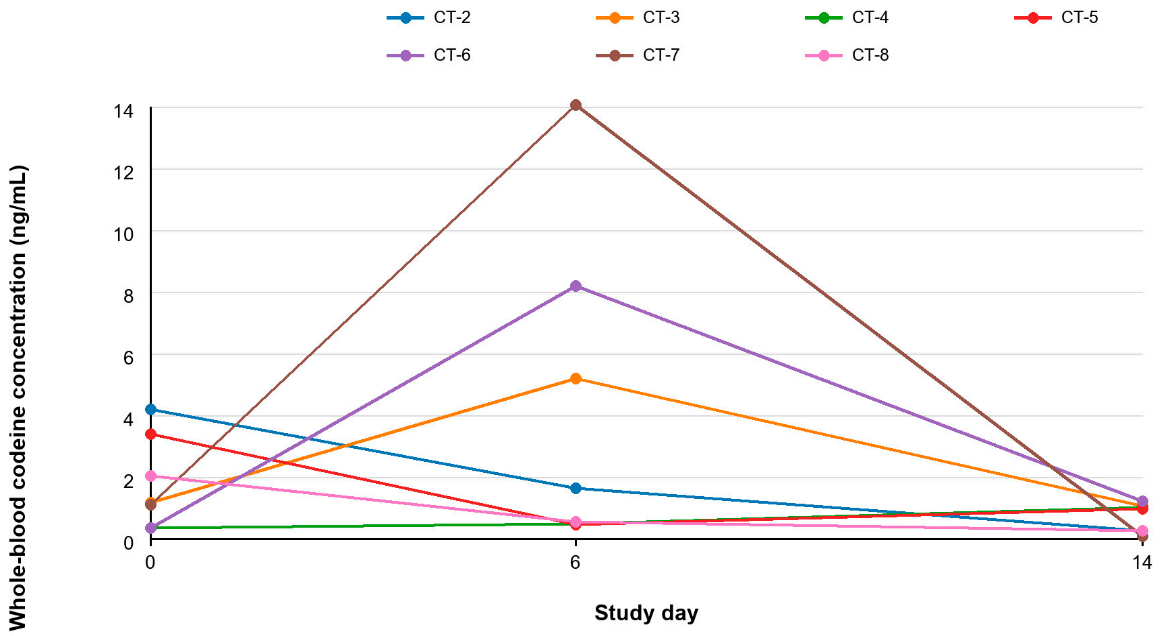

Figure S2. Individual instrument-reported whole-blood codeine profiles in the combined codeine plus testosterone undecanoate group (CT), n = 7 animals with available values on Days 0, 6, and 14. Concentrations are ng/mL. One CT measurement (CT7 Day 14, 0.081188 ng/mL) was below the verified 0.25 ng/mL LOQ but remained above the 0.07 ng/mL LOD and is displayed as reported.

Table S1. Available individual whole-blood codeine concentrations

Group	Animal	Day	Codeine (ng/mL)	Analytical status
C	2	0	1.652260	At/above LOQ
C	2	6	0.523216	At/above LOQ
C	2	14	2.915065	At/above LOQ
C	3	0	1.298960	At/above LOQ
C	3	6	1.345759	At/above LOQ
C	3	14	4.053037	At/above LOQ
C	4	0	4.221959	At/above LOQ
C	4	6	1.016913	At/above LOQ
C	4	14	0.939475	At/above LOQ
C	5	0	4.705022	At/above LOQ
C	5	6	2.121543	At/above LOQ
C	5	14	1.952177	At/above LOQ
C	6	0	1.989909	At/above LOQ
C	6	6	2.413102	At/above LOQ
C	6	14	1.566322	At/above LOQ
C	7	0	1.946811	At/above LOQ
C	7	6	0.968317	At/above LOQ

Group	Animal	Day	Codeine (ng/mL)	Analytical status
C	7	14	3.241254	At/above LOQ
C	8	0	0.656914	At/above LOQ
C	8	6	0.983848	At/above LOQ
C	8	14	1.084318	At/above LOQ
CT	2	0	4.194703	At/above LOQ
CT	2	6	1.622673	At/above LOQ
CT	2	14	0.252690	At/above LOQ
CT	3	0	1.163084	At/above LOQ
CT	3	6	5.192603	At/above LOQ
CT	3	14	1.046976	At/above LOQ
CT	4	0	0.353638	At/above LOQ
CT	4	6	0.475915	At/above LOQ
CT	4	14	1.000829	At/above LOQ
CT	5	0	3.399278	At/above LOQ
CT	5	6	0.459451	At/above LOQ
CT	5	14	0.972584	At/above LOQ
CT	6	0	0.339435	At/above LOQ
CT	6	6	8.195449	At/above LOQ
CT	6	14	1.204357	At/above LOQ
CT	7	0	1.091319	At/above LOQ
CT	7	6	14.066489	At/above LOQ
CT	7	14	0.081188	Below LOQ; above LOD
CT	8	0	2.027914	At/above LOQ
CT	8	6	0.550903	At/above LOQ
CT	8	14	0.259280	At/above LOQ

No values were available for C1 or CT1 because whole-blood sample volume was insufficient for these measurements. These blanks were not converted to zero or imputed.

Table S2. Available individual plasma total-testosterone concentrations

Group	Animal	Day	Testosterone (pg/mL)
C	1	14	59.573018
C	2	14	73.389608
C	3	0	555.902506
C	3	6	356.460212
C	3	14	161.564327
C	4	6	272.678780
C	4	14	133.286185
C	5	0	686.600367

Group	Animal	Day	Testosterone (pg/mL)
C	5	14	66.455801
C	6	6	262.438265
C	6	14	122.666438
C	7	0	369.608432
C	7	6	577.128437
C	7	14	101.452278
C	8	0	808.634718
C	8	6	306.525512
C	8	14	76.872354
CT	1	0	286.270915
CT	1	6	186.190930
CT	1	14	248.723088
CT	2	6	136.825496
CT	2	14	56.154531
CT	3	0	245.283503
CT	3	6	143.901438
CT	3	14	87.370735
CT	4	0	238.391561
CT	4	6	269.269676
CT	4	14	269.269676
CT	5	14	97.925229
CT	6	0	349.859825
CT	6	6	405.409679
CT	6	14	112.051321
CT	7	14	150.972102
CT	8	0	669.145081
CT	8	6	214.139272
CT	8	14	245.283503
S	1	0	196.698532
S	1	14	52.753616
S	2	0	241.839651
S	2	14	80.364130
S	3	14	49.372214
S	4	0	712.579392
S	4	6	136.825496
S	4	14	112.051321
S	5	0	363.043055
S	5	14	63.007312
S	6	0	97.925229
S	6	6	245.283503
S	6	14	66.455801
S	7	0	59.573018
S	7	14	101.452278
S	8	0	69.917014
S	8	6	140.364004

Group	Animal	Day	Testosterone (pg/mL)
S	8	14	66.455801
T	1	0	1020.630455
T	1	6	466.057120
T	1	14	203.686005
T	2	0	509.825300
T	2	14	147.437548
T	3	0	497.397009
T	3	6	333.281660
T	3	14	80.364130
T	4	0	299.791839
T	4	14	210.658666
T	5	0	689.498909
T	5	14	175.653926
T	6	14	97.925229
T	7	0	645.697530
T	7	14	175.653926
T	8	6	221.088737
T	8	14	336.606147

Available n on Days 0/6/14: S 7/3/8; C 4/5/8; T 6/3/8; CT 5/6/8. Blank plasma measurements in the source workbook were not converted to zero or imputed.

Data-handling note

This supplement reproduces the numerical values available in the supplied Excel workbook. Blank cells caused by insufficient sample volume were not converted to zero or imputed. Codeine was measured in whole blood. With the verified LOQ of 0.25 ng/mL and LOD of 0.07 ng/mL, only CT7 Day 14 (0.081188 ng/mL) was below the LOQ while remaining above the LOD; it is retained in Table S1 for transparency. The primary codeine model used all instrument-reported numerical values, and the post hoc sensitivity analysis excluding this observation is reported in the main manuscript. Total testosterone was measured in plasma. Eighteen instrument-generated plasma testosterone values were below the stated lower reporting boundary of 100 pg/mL and are reproduced as reported; the corresponding sensitivity analyses are described in the main manuscript.
